# Aripiprazole-Cyclodextrin Binary Systems for Dissolution Enhancement: Effect of Preparation Technique, Cyclodextrin Type and Molar Ratio

**Published:** 2013-12

**Authors:** Shaimaa M. Badr-Eldin, Tarek A. Ahmed, Hatem R Ismail

**Affiliations:** 1 Department of Pharmaceutics and Industrial Pharmacy, Faculty of Pharmacy, King Abdulaziz University, Jeddah, KSA; 2 Department of Pharmaceutics and Industrial Pharmacy, Faculty of Pharmacy, Cairo University, Cairo Egypt; 3 Department of Pharmaceutics and Industrial Pharmacy, Faculty of Pharmacy, Al-Azhar University, Cairo, Egypt

**Keywords:** Aripiprazole, Binary systems, Cyclodextrin, * In vitro* dissolution phase-solubility

## Abstract

***Objective(s):*** The aim of this work was to investigate the effect of the natural and the chemically modified form of cyclodextrins namely; β-cyclodextrin (β-CD) and hydroxypropyl-β-cyclodextrin (HP-β-CD) respectively on the solubility and dissolution rate of aripiprazole; an antipsychotic medication showing poor aqueous solubility.

***Materials and Methods:*** Phase solubility of aripiprazole with the studied CDs and the complexation efficiency values (CE) which reflect the solubilizing power of the CDs towards the drug was performed. Solid binary systems of aripiprazole with CDs were prepared by kneading, microwave irradiation and freeze-drying techniques at 1:1 and 1:2 (drug to CD) molar ratios. Drug-CD physical mixtures were also prepared in the same molar ratios for comparison. The dissolution of aripiprazole-binary systems was carried out to select the most appropriate CD type, molar ratio and preparation technique.

***Results:*** Phase solubility study indicated formation of higher order complexes and the complexation efficiency values was higher for HP-β-CD compared to β-CD. Drug dissolution study revealed that aripiprazole dissolution was increased upon increasing the CD molar ratio and, the freeze-drying technique was superior to the other studied methods especially when combined with the HP-β-CD. The cyclodextrin type, preparation technique and molar ratio exhibited statistically significant effect on the drug dissolution at *P*≤ 0.05.

***Conclusion:*** The freeze-dried system prepared at molar ratio 1:2 (drug: CD) can be considered as efficient tool for enhancing aripiprazole dissolution with the possibility of improving its bioavailability.

## Introduction

Several approaches have been applied to improve the bioavailability of poorly water-soluble drugs. These approaches include; chemical and physical modifications. The former involves incorporating of water solubilizing groups into the drug structure such as the alcohol, amine, amide, carboxylic acid, sulfonic acid and phosphate groups. Pinnamaneni *et al* reported the incorporation of acidic and basic groups, which form salts that would result in a wider range of dosage forms for the final product (1). Physical modification includes particle size reduction (2), modification of the crystal structure (1), drug dispersion in carriers and complexation (3, 4). 

Cyclodextrins, a group of structurally related natural products, formed during bacterial digestion of cellulose, are crystalline, water soluble, cyclic, oligosaccharides, consist of glucose monomers arra-nged in a donut shape ring molecules. The most common cyclodextrins are alpha, beta, and gamma types having six (α), seven (β), or eight (γ) anhydro-glucose units in the ring structure. Cyclodextrins are widely used as "molecular cages" in the pharmace-utical, agrochemical, food and cosmetic industries (5). Among different cyclodextrins, the beta and its hydroxyalkylated derivative, 2-hydroxypropyl-β-CD (HP-β-CD) are widely used in pharmaceutical industry as complexing agents to increase the aqueous solubility of poorly soluble drugs and to increase their bioavailability and stability (6).

Several methods have been published for drug-CD inclusion complex formation including physical blending (7), kneading (8-10), co-precipitation technique (11, 12), solution/solvent evaporation (13), neutralization precipitation (14), milling/co-grinding (15), atomization/spray drying (16), Lyophilization/Freeze-drying (17), microwave irradiation (18-20), and supercritical antisolvent technique (21, 22).

Aripiprazole; 7-[4-[4-(2, 3-Dichlorophenyl) piper-azin-1-yl] butoxy]-3, 4-dihydro-1H-quinolin-2-one, is an atypical antipsychotic medication used for the treatment of schizophrenia. The drug received FDA approval for the treatment of acute manic and mixed episodes associated with bipolar disorder. Aripip-razole appears to mediate its antipsychotic effects primarily by partial agonist at the D_2_ receptor (23). Aripiprazole shows poor aqueous solubility that may lead to highly variable blood levels, and irreproduc-ible clinical response. 

Thus, the aim of this work was to enhance the dissolution of aripiprazole utilizing the approach of complexation with cyclodextrins. Phase-solubility technique was used to study the interaction of aripiprazole with β-cyclodextrin and its hydroxyl-propyl derivative in the solution state. Solid aripiprazole-cyclodextrin binary systems were prepared using different preparation methods. *In vitro* dissolution studies of all the prepared systems were carried out to investigate the effect of molar ratio, cyclodextrin type and preparation method on aripiprazole dissolution profile. 

## Materials and Methods

***Materials***

Aripiprazole, MW = 448, was purchased from Dr Reddy's Laboratories Ltd (Hyderabad, India). Beta cyclodextrin (β-CD), MW = 1135 was kindly supplied by International Specialty Products Co. (ISP, Germany). 2-Hydroxypropyl beta cyclodextrin (HP-β-CD), MW = 1460 & DS = 0.8 was supplied from Sigma-Aldrich, packed in Germany. Absolute ethyl alcohol and glacial acetic acid were purchased from Fluka, Germany.

***Phase solubility study***

The effect of β-CD and HP-β-CD on the solubility of aripiprazole was investigated according to the phase solubility technique established by Higuchi and Connors (24). Excess amounts of aripiprazole (25 mg) were added to 10 ml of either distilled water or aqueous solutions containing increasing concentrations of the previously mentioned cyclodextrins (ranging from 2 to 20 mM) in a series of 20 ml glass stoppered vials. The obtained suspensions were shaken at 37 ± 0.5°C using thermostatically controlled shaking water bath (Model 1083; GLF Corp., Burgwedel, Germany) for 7 days. A preliminary experiment was conducted to determine the time needed to achieve equilibrium. Aliquots were withdrawn and filtered through a Millipore membrane filter (0.45 μm pore size, Type μstar LB; Costar Corp., Cambridge, USA). The filtered solutions were analyzed spectrophotometrically for aripiprazole content by measuring the absorbance at λmax 256 nm against blank solutions containing the same concentrations of cyclodextrins (25). Each experiment was carried out in triplicate. Phase solubility diagrams were obtained by plotting the molar concentration of the solubilized aripiprazole versus the molar concentrations of the cyclodextrins used. The apparent stability constants (Ks) were estimated from the straight line of the phase solubility diagrams according to the following equation of Higuchi and Connors (24):

K_s_ = slope / S_0_ (1-slope)

Where So represents the drug solubility in absence of cyclodextrins (the intercept of the phase solubility diagram).

The complexation efficiency (CE), which represents the solubilizing efficiency of the cyclodextrins for the drug, was also calculated from the slope of the phase solubility profile as the ratio of the complex to free cyclodextrin concentration, according to the following equation (26):

CE = S_0_ K1:1 = [drug-CD]/[CD] = slope / 1-slope

Where [drug-CD] is the concentration of the drug-CD complex and [CD] is the concentration of the free cyclodextrin.

***Preparation of aripiprazole-cyclodextrin binary solid systems***

Solid binary systems of aripiprazole with β-CD and HP-β-CD were prepared in molar ratios of 1:1 and 1:2 (drug to CD). The solid binary systems were prepared by kneading, microwave irradiation and freeze-drying techniques. Physical mixtures were also prepared in the same molar ratios for comparison.

***Physical mixtures***

Physical mixtures of aripiprazole and each of the cyclodextrins were prepared by thoroughly mixing the two components in a mortar for 30 min.

***Kneading method***

The required amounts of aripiprazole and cyclo-dextrin were accurately weighed, transferred to a mortar and triturated with small volume of ethanol-water (50:50, v/v) solution (27). The slurry obtained was kneaded for 30 min and then dried under vacuum at room temperature in presence of calcium chloride as a dehydrating agent.

***Microwave irradiation method***

The required amounts of aripiprazole and cyclo-dextrin were accurately weighed. A homogenous paste was prepared by mixing the studied cyclodextrins and the drug with minimum amounts of solvents (ethanol: water, 1:1 v/v) in a mortar. The paste formed was reacted for 90 s at 60°C using a microwave oven (EM-G A, Sanyo, Japan) (18).

***Freeze-drying***

Lyophilization monophase solution method was used to prepare the drug-CD binary phase (27). Appropriate quantities of cyclodextrin and aripiprazole were dissolved in distilled water and glacial acetic acid, respectively and the resulted solutions were mixed by stirring. The clear monophase solution was frozen at -20°C, and subsequently freeze-dried for 24 hours at -50°C using a Freeze-dryer (Novalyphe-NL 500; Savant Instruments Corp., Holbrook, NY, USA).

Physicochemical characterization 

***Infrared spectroscopy***

The pure drug, pure cyclodextrins and their binary mixtures were subjected to infrared (IR) characterization to predict any possible interaction. Shimadzu differential scanning calorimeter (DSC-50, Shimadzu, Japan) was used in which each sample was mixed with KBr powder and compressed into transparent disc under high pressure. The resulting discs were then tested within the range 4000–500 cm^−1^. 

***Differential scanning calorimetry***

All samples used in the IR spectroscopy were also subjected to differential scanning calorimetry (DSC) characterization. Encapsulation of these samples into flat-bottom aluminum pans with crimped-on lids were performed before placing the samples into a Shimadzu DSC TA-50 ESI DSC apparatus (Tokyo, Japan). The scanning speed was 10°C/min and the flow rate was 30 mL/min.

***In vitro dissolution studies of aripiprazole-CD binary systems***

The dissolution of pure aripiprazole and the prepared binary systems was performed using the USP Dissolution Tester, paddle type, Apparatus IІ, (Erweka Corp., Germany). The paddle speed was 100 rpm and the study was performed in 400 ml distilled water, at a temperature of 37 ± 0.5°C. The paddle was placed at 2.5 cm from the bottom of the vessel. A drug sample of 10 mg, its equivalent of the complexes or the physical mixtures was spread on the surface of the dissolution medium. At appro-priate time intervals of 5, 10, 15, 20, 30, 45 and 60 minutes, aliquots each of 5 ml were withdrawn from the dissolution medium through millipore filter (pore size 0.45 μm) and replaced with an equivalent amount of the fresh dissolution medium in order to maintain the volume in the vessel constant. The filtered solutions were analyzed spectrophoto-metrically for aripiprazole content by measuring the absorbance at λ_max_ 256 nm against distilled water as a blank. Each experiment was done in triplicate. The extent of dissolution after 60 minutes (DP_60_ %), dissolution efficiency at 60 minutes (DE_60_, %) and the initial dissolution rate (IDR_5min_, µg/min) were used as tools to evaluate the drug dissolution profile.

$\mathrm{Dissolution efficiency}(\mathrm{DE})=\int_{0}^{t}\mathrm{y.dt}/y_{100}t^{*}100$   (28)

**Figure 1:**
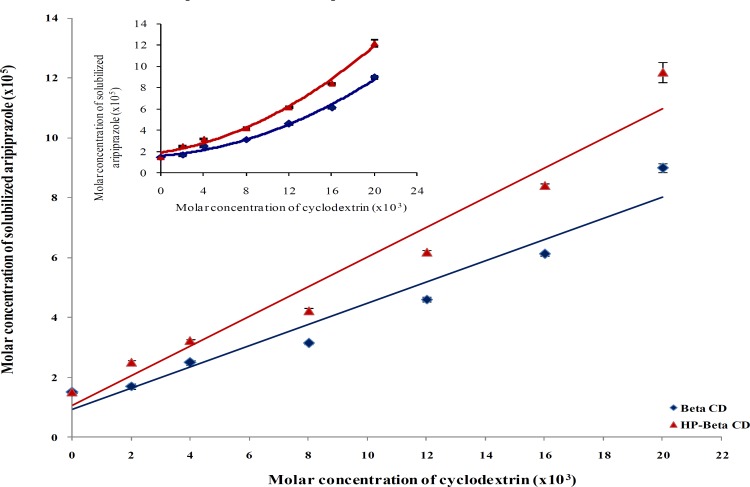
Phase solubility diagrams of aripiprazole with β-CD and HP- β-CD in distilled water at 37 ± 0.5°C

**Where the integral is the area under dissolution curve up to time t and y**_100_ **is the area of the rectangle described by 100 % dissolution at the same time.**

The DE_60_ of the binary systems was statistically analyzed using two-way ANOVA to test the significance of the effects of the preparation method, cyclodextrin type and molar ratio at *P* 0.05. Statistical analysis was performed using StatView^®^ software (Abacus Inc, version 4.75).

## Results

***Phase solubility studies***

Phase solubility diagrams of aripiprazole with the studied cyclodextrins in distilled water at 37 ± 0.5°C are graphically illustrated in Figure 1. The values of the stability constants (K_s_) were calculated and found to be 379.028 and 464.740 M^-1^ for β-CD and HP-β-CD, respectively whereas, the complexation efficiency (CE) values were computed and found to be 0.00356 and 0.00497 for β-CD and HP-β-CD, respectively.

***Physicochemical characterization***

**Figure 2:**
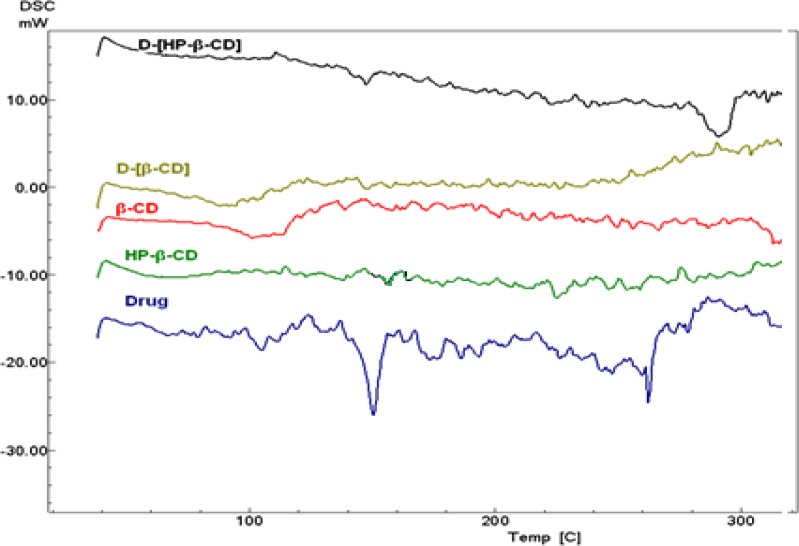
Differential Scanning Calorimetry thermograms of pure aripiprazole, HP β-CD, β-CD, and their physical mixtures

The DSC curve of aripiprazole showed a melting endothermic point at around 140°C as shown in Figure 2. Mixing the drug with the studied cyclodextrins resulted in partial disappearance or decrease in the intensity of the drug melting endothermic peak that could be attributed to complexation. Such effect was obvious in the mixtures prepared by kneading, microwave irradiation and freez-drying (curves not shown).

**Figure 3:**
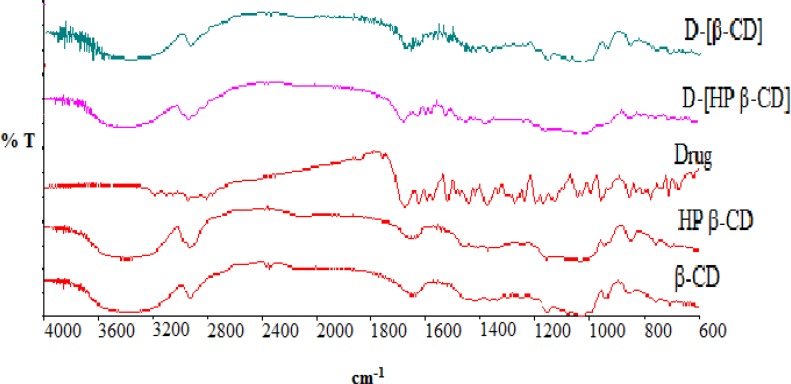
Infra Red spectra of pure aripiprazole, HP β-CD, β-CD, and their physical mixtures

**Figure 4:**
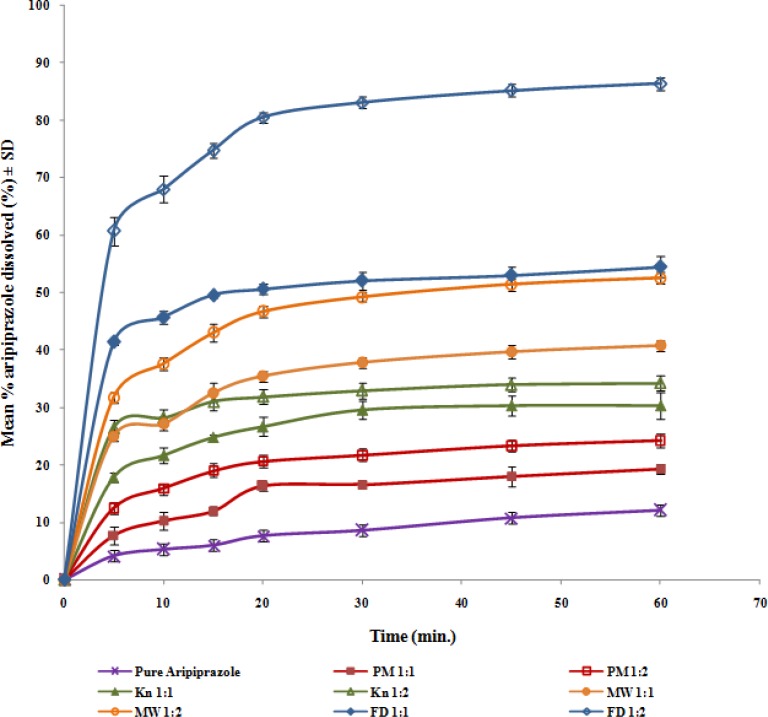
Dissolution profiles of aripiprazole from aripiprazole-HP-β-CD binary systems in distilled water at 37 ± 0.5°C

**Figure 5:**
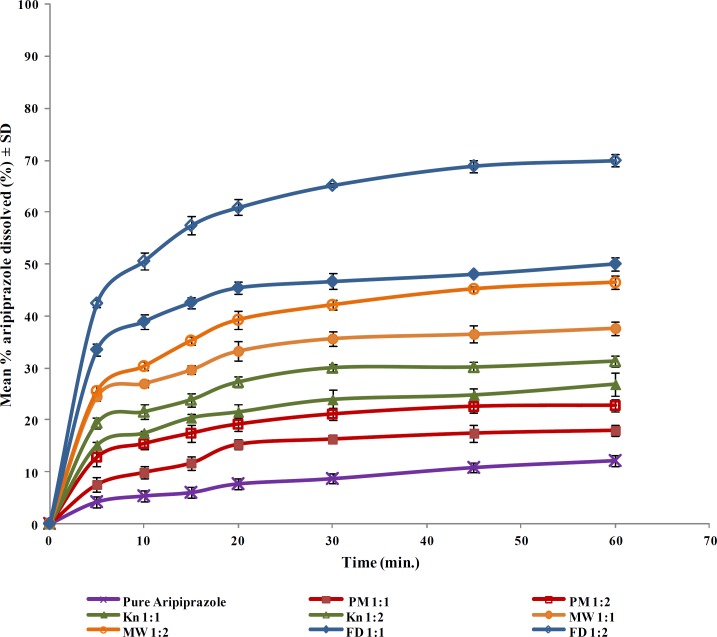
Dissolution profiles of aripiprazole from aripiprazole-β-CD binary systems in distilled water at 37 ± 0.5°C

**Table 1:** Dissolution parameters of aripiprazole from different drug-cyclodextrin prepared binary systems

**AR-CD** **system**	**Extend of dissolution after** **60 min (DP60 %)**				**Dissolution efficiency** **(DE %)**				**Initial dissolution rate** **(IDR **_5min _ **, ** **µ** **g/min)**			
	**PM**	**KN**	**MW**	**FD**	**PM**	**KN**	**MW**	**FD**	**PM**	**KN**	**MW**	**FD**
**AR-** ***β-CD*** **1:1** * **1:2** *	17.93 ± 1.04	26.86 ±2.17	37.66 ± 1.23	50.06 ±1.23	14.30 ± 0.92	21.65 ±0.17	32.19 ±1.28	43.27 ±0.30	1.51 ±0.28	3.03 ±0.13	4.91 ±0.15	6.71 ±0.25
	22.73 ± 1.23	31.26 ±1.04	46.46 ±1.23	69.99 ±1.13	19.05 ± 0.01	26.50 ±0.65	38.39 ±0.64	59.72 ±0.02	2.56 ±0.34	3.88 ±0.21	5.11 ±0.21	8.49 ±0.13
**AR-HP** *** β-CD*** **1:1** * **1:2** *	19.26 ±0.85	30.34 ±2.38	40.80 ± 0.94	54.53 ±1.89	14.90 ±0.61	26.12 ±1.25	34.41 ±0.66	48.66 ±1.25	1.55 ±0.30	3.55 ±0.18	5.21 ±0.19	8.29 ±0.11
	24.26 ± 1.13	34.26 ±1.32	52.59 ±0.85	88.39 ±1.13	19.95 ± 0.10	30.77 ±1.25	44.91 ± 0.88	76.46 ±0.45	2.51 ±0.19	5.29 ±0.28	6.35 ±0.11	12.15 ±0.51

* drug to polymer molar ratio. Data presented as mean ± SD, n=3

***In vitro dissolution studies***

The dissolution profiles of aripiprazole from its solid drug-CD binary systems are graphically illustrated in Figures 4 & 5. A quantity equivalent to 10 mg aripiprazole was added to the dissolution medium, based on the percentage drug content determined for each product.

In order to investigate the effect of the different variables (types of CD, molar ratios and preparation techniques) on the dissolution of the drug from its binary systems, the extent of dissolution after 60 min (DP_60_), the dissolution efficiency and the initial dissolution rate after 5 min (IDR_5min_) were calculated, data are presented in Table 1.

## Discussion

It was evident that cyclodextrins had a pronounced effect in the solubility of aripiprazole. This could be attributed to the formation of inclusion complexes (24). However, other interactions capable of solubilizing insoluble drugs via non-inclusion complexation or micelle-like structure may be involved (29). The solubilizing power of the invest-tigated cyclodextrins; natural and chemically modified form; towards aripiprazole was higher with HP-β-CD than the corresponding β-CD which is similar to previous finding observed with other drugs solubilized with β-CD and its derivatives (29-31). 

Data obtained from the phase solubility diagram was used to estimate the solubility coefficient of determination (r^2^), which differentiates between A_P_ and A_L _types. The results revealed that, the r^2 ^values were < 0.990 (0.948 and 0.957 for β-CD and HP-β-CD, respectively) which is to be classified as A_P_ type curves according to Arima *et al* (32). This assumes the formation of drug-polymer inclusion complexes of higher order characterized by stepwise binding constants. However, they can also indicate the formation of complex aggregates that are capable of solubilizing additional amounts of the guest via non inclusion complexation or micelle–like structure (29, 33). Similar results for the increase in solubility of some drugs as a function of increasing cyclodextrins concentrations were reported for glipizide, and repaglinide (34, 35).

Ramnik *et al* reported that, the effect of cyclodextrins on the drug DSC thermogram is observed as broadening, shifting and appearance of new peaks or disappearance of certain peaks (36). In our DSC results, the drug characteristic peaks were disappeared and shifted upon mixing the drug with β-CD, HP β-CD respectively. It was previously stated that, for FTIR spectrum, the cyclodextrin bands often change only slightly upon complexation and if the fraction of the guest molecules encapsulated in the complex is less than 25%, bands which could be assigned to the included part of the guest molecules are easily masked by the bands of the spectrum of cyclodextrin (36). The FTIR spectrum of aripiprazole shows a characteristic peaks at 3193 cm^-1^(N-H stretching), 1678 cm^-1^ (-C=O stretching), and 1138 cm^-1^ (aromatic C-Cl stretching). The FTIR spectra of the pure CDs illustrated intense broad absorption bands at 3500–3300 cm^_1^ corresponding to the free –OH stretching. The vibration of –CH and –CH_2_ groups appeared in the region 2950– 2600 cm^_1^ and the shorter band appeared in the region 1650–1640 cm^_1^ could be related to the hydrated bonds within cyclodextrin molecules. Another large band, assigned to the C– O–C stretching vibration, displaying distinct sharp peaks occurred between 1200 and 1030 cm^_1^ (37). Drug-cyclodextrins complexation almost keeps the characteristic peaks for the drug and the studied cyclodextrin as illustrated in Figure (3). Only a slight modification in aripiprazole characteristic peak at 1138 cm-1 corresponding to aromatic C-Cl stretching was observed. Both DSC and FT-IR spectrum confirmed the complexation between the drug and the studied polymers. 

Statistical analysis of the DE_60_ data for the prepared aripiprazole-CD systems was performed using two-way ANOVA. The results revealed the presence of significant differences among the different cyclodextrin types (β-CD, HP-β-CD), preparation methods (PM, KN, MW and FD) and molar ratios (1:1 and 1:2, drug to CD) at *P* 0.05. The computed F-values indicated that the dissolution profile of the drug from its binary cyclodextrin systems was dependent on the different factor as follows: preparation method > molar ratio > CD type.

It was evident that, pure aripiprazole powder showed slow dissolution rate with cumulative % dissolved of 12.1% after 60 min under the specified dissolution conditions. Owing to its hydrophobic property, the drug contact with the dissolution medium is prevented and consequently, its dis-solution is hindered. The slight enhancement of drug dissolution when physically mixed with the studied cyclodextrins could be attributed to the local solubilization action of the carrier operating in the microenvironment or the hydrodynamic layer surrounding drug particles in the early stages of the dissolution process or due to the surfactant-like properties of cyclodextrins that improve the drug wettability by reducing the interfacial tension between the water insoluble drug particles and the dissolution medium, thus improving the wettability and dissolution of the drug (31).

The kneaded products showed slightly more enhancement in aripiprazole dissolution compared to the physical mixtures. Murthy and Sowjanya (12) reported faster dissolution rate for carvedilol inclusion complexes with HP- *β*-cyclodextrins obtained by Kneading method at 1:2 molar ratios than the corresponding complexes prepared by physical mixing and solvent evaporation methods. Similar observations have been reported by other authors with other drugs (8, 38). The observed slight increment in drug dissolution compared to the physical mixtures is probably due to the increase in the drug-carrier contact surface owing to increased mechanical treatment (8). However, the improve-ment of dissolution is limited because the inter-actions between the drug and the cyclodextrin might be hindered due to employment of semisolid medium (11).

Binary systems prepared by microwave irradi-ation showed more enhancements in the dissolution of aripiprazole compared to the physical mixtures and kneaded products which could be ascribed to the enhanced interaction between the drug and the cyclodextrins by virtue of the energy of microwave irradiation. Inclusion complexes prepared with β –CD and HP- β -CD by microwave irradiation method showed highest enhancement in the solubility of ziprasidone than that prepared by kneading or coprecipitation methods (19). Similar dissolution enhancement was observed for carvedilol-β-CD binary systems prepared by microwave-irradiation (18). The time of microwave irradiation was selected on the basis of preliminary study where different times ranging from 30 to 120 sec were applied for the preparation of the binary systems. The optimum irradiation timing was 90 sec. Higher irradiation time leads to reduction in dissolution times that might be due to increased bond interaction between the drug and the cyclodextrin (20). 

The freeze-dried systems showed marked increase in aripiprazole dissolution compared to the other methods. This marked dissolution enhance-ment could be attributed mainly to the formation of soluble inclusion complexes of the drug with the cyclodextrins and increased energy of the drug due to reduction of the crystallinity following comple-xation (39). Additional theory stated that the marked increase in the dissolution rate might be due to the formation of solid solution of the drug in the freeze-dried products as a result of the complete inclusion of the drug into the cyclodextrin cavities and the particle size is reduced to the molecular size when the carrier brought the drug into the dissolution medium, leading to fast dissolution (40). The observ-ed lower increment in drug dissolution from β-CD freeze-dried systems compared to HP-β-CD could result from the lower water solubility of β-CD (9).

It is worthy to mention that, glacial acetic acid was able to dissolve aripiprazole effectively. It is considered as one of the good solvents for freez-drying processes as it is volatile and miscible with water in all proportions with consequent enhance-ment in the encapsulation of the drug into the cyclodextrin cavity upon evaporation. Several previous studies used glacial acetic acid as a solvent to improve cyclodextrin complexation with hydro-phobic drugs (37, 41, 42).

It was clear that the drug dissolution was enhanced upon increasing the cyclodextrin pro-portion. Physical mixtures showed the least effect for the molar ratio since the enhancement in dissolution is mainly due to the wetting effect of the cyclo-dextrins, to which cyclodextrins contribute to an equal extent, with their different molar ratios (11). Conversely, the most pronounced effect for the molar ratio was observed for the freeze-dried products due to better dispersion and/or inclusion of the drug with increasing the cyclodextrin molar ratio during preparation. Balata *et al* reported the increase in both solubility and dissolution of ketoconazole prepared by solid dispersion and inclusion complexes in β-cyclodextrin as the molar ratio increased (43). Similar results were also obtained with the spray-dried complexes of FPFS-410 (38). Compared to previously published work for the enhancement of aripiprazole solubility by comple-xation with (2-hydroxy) propyl-β-cyclodextrin utilizing the spray drying technique (44), we studied the effect of both natural and chemically modified form of cyclodextrins in which the latter was superior in its results, also our work focused on utilization of more simple and cheap techniques namely; kneading, microwave irradiation and freeze-drying method.

The effect of CD type was also one of the important variables affecting the drug dissolution from the prepared solid binary systems. The systems prepared using HP-β-CD showed marked better enhancement in aripiprazole dissolution compared to those prepared using the parent β-CD, especially when using the freeze-drying technique. This result could be explained on the basis of greater water solubility, better wetting ability and higher complexing power of CD derivatives towards the drug in the solid state (45, 46). Similar results have been also reported for improving the solubility and dissolution of poorly water soluble drugs on the same basis but utilizing the solid dispersion technique. Indomethacin dissolution rate has been improved utilizing PVP K30 and isomalt (GALEN IQ 990) using the solvent evaporation technique and the hot melt method (47).

## Conclusion

From the above results, it is possible to conclude that HP-β-CD showed better solubility and dissolution enhancement for aripiprazole compared to β-CD. This enhancement was obviously demonstrated when preparing drug-CD binary system in a 1:2 molar ratio using the freeze-drying technique. These conditions showed an initial burst effect of more than 60 % in the first 5 min and more than 80% dissolution within 30 min. Therefore, the aforementioned system can be considered as efficient tool for enhancing the dissolution of aripiprazole with the possibility of improving the bioavailability and thus reducing the dose of the drug.
